# Scope of Artificial Intelligence in Gastrointestinal Oncology

**DOI:** 10.3390/cancers13215494

**Published:** 2021-11-01

**Authors:** Hemant Goyal, Syed A. A. Sherazi, Rupinder Mann, Zainab Gandhi, Abhilash Perisetti, Muhammad Aziz, Saurabh Chandan, Jonathan Kopel, Benjamin Tharian, Neil Sharma, Nirav Thosani

**Affiliations:** 1 Department of Internal Medicine, The Wright Center for Graduate Medical Education, 501 S. Washington Avenue, Scranton, PA 18505, USA; 2 Department of Medicine, John H Stroger Jr Hospital of Cook County, 1950 W Polk St, Chicago, IL 60612, USA; syedaliamir.sherazi@cookcountyhhs.org; 3 Department of Medicine, Saint Agnes Medical Center, 1303 E. Herndon Ave, Fresno, CA 93720, USA; rupindrmann@yahoo.com; 4 Department of Medicine, Geisinger Wyoming Valley Medical Center, 1000 E Mountain Dr, Wilkes-Barre, PA 18711, USA; drzainabgandhi@gmail.com; 5 Division of Interventional Oncology & Surgical Endoscopy (IOSE), Parkview Cancer Institute, 11050 Parkview Circle, Fort Wayne, IN 46845, USA; abhilash.perisetti@gmail.com (A.P.); neil.sharma@parkview.com (N.S.); 6 Department of Gastroenterology and Hepatology, University of Toledo Medical Center, 3000 Arlington Avenue, Toledo, OH 43614, USA; marajani@hotmail.com; 7 Division of Gastroenterology and Hepatology, CHI Health Creighton University Medical Center, 7500 Mercy Rd, Omaha, NE 68124, USA; saurabhchandan@gmail.com; 8 Department of Medicine, Texas Tech University Health Sciences Center, 3601 4th St, Lubbock, TX 79430, USA; jonathan.kopel@ttuhsc.edu; 9 Department of Gastroenterology and Hepatology, The University of Arkansas for Medical Sciences, 4301 W Markham St, Little Rock, AR 72205, USA; btharian@uams.edu; 10 Division of Gastroenterology, Hepatology & Nutrition, McGovern Medical School, UTHealth, 6410 Fannin, St #1014, Houston, TX 77030, USA; nirav.thosani@uth.tmc.edu

**Keywords:** artificial intelligence, colorectal cancer, gastrointestinal cancer, hepatocellular cancer, pancreaticobiliary cancer, gastric cancer, esophageal cancer

## Abstract

**Simple Summary:**

Gastrointestinal cancers cause over 2.8 million deaths annually worldwide. Currently, the diagnosis of various gastrointestinal cancer mainly relies on manual interpretation of radiographic images by radiologists and various endoscopic images by endoscopists. Artificial intelligence (AI) may be useful in screening, diagnosing, and treating various cancers by accurately analyzing diagnostic clinical images, identifying therapeutic targets, and processing large datasets. The use of AI in endoscopic procedures is a significant breakthrough in modern medicine. Although the diagnostic accuracy of AI systems has markedly increased, it still needs collaboration with physicians. In the near future, AI-assisted systems will become a vital tool for the management of these cancer patients.

**Abstract:**

Gastrointestinal cancers are among the leading causes of death worldwide, with over 2.8 million deaths annually. Over the last few decades, advancements in artificial intelligence technologies have led to their application in medicine. The use of artificial intelligence in endoscopic procedures is a significant breakthrough in modern medicine. Currently, the diagnosis of various gastrointestinal cancer relies on the manual interpretation of radiographic images by radiologists and various endoscopic images by endoscopists. This can lead to diagnostic variabilities as it requires concentration and clinical experience in the field. Artificial intelligence using machine or deep learning algorithms can provide automatic and accurate image analysis and thus assist in diagnosis. In the field of gastroenterology, the application of artificial intelligence can be vast from diagnosis, predicting tumor histology, polyp characterization, metastatic potential, prognosis, and treatment response. It can also provide accurate prediction models to determine the need for intervention with computer-aided diagnosis. The number of research studies on artificial intelligence in gastrointestinal cancer has been increasing rapidly over the last decade due to immense interest in the field. This review aims to review the impact, limitations, and future potentials of artificial intelligence in screening, diagnosis, tumor staging, treatment modalities, and prediction models for the prognosis of various gastrointestinal cancers.

## 1. Introduction

Artificial intelligence is described as the intelligence of machines compared to natural human intelligence. It is a computer science field dedicated to building a machine that simulates the cognitive functions of humans, such as learning and problem solving [1,2]. Recent advances in artificial intelligence technologies have been developed due to technical advances in deep learning technologies, support vector machines, and machine learning, and these advanced technologies have played a significant role in the medical field [3,4,5]. Virtual and physical are the two main branches of AI in the medical field. Machine learning (ML) and deep learning (DL) are two branches of the virtual branch of AI. Convolutional neural networks (CNN), an essential deep neural network, represent a multilayer artificial neural network (ANN) useful for image analyses. The physical branch of AI includes medical devices and robots [6,7].

As per the WHO report, nearly 5 million new gastrointestinal, pancreatic, and hepatobiliary cancers were recorded worldwide in 2020. Gastrointestinal cancers include esophageal, colorectal (colon and rectum), and gastric cancer. Colorectal cancer (CRC) is the most common cancer of all gastrointestinal cancers. Overall, CRC is the second in terms of mortality and third in incidence after breast and lung cancers globally [8]. Although there are significant advances in diagnostics, including predictive and prognostic biomarkers and treatment approaches for gastrointestinal, pancreatic, and hepatobiliary cancers, there is still high potential to improve further for better clinical outcomes and fewer side effects [6,9]. The data from advanced imaging modalities (including advanced endoscopic techniques with the addition of AI) with high accuracy, novel biomarkers, circulating tumor DNA, and micro-RNA can be beyond human interpretation. In the clinical setting, various diagnostic methods (endoscopy, radiologic imaging, and pathologic techniques) using AI, including imaging analysis, are needed [10,11,12,13,14,15]. In this narrative review, we discussed the application of AI in diagnostic and therapeutic modalities for various gastrointestinal, pancreatic, and hepatobiliary cancers.

## 2. Esophageal Cancers

Esophageal cancer, comprising esophageal adenocarcinoma and esophageal squamous cell carcinoma (ESCC), is the ninth most common cancer globally by incidence and sixth by cancer mortality, with an estimated over 600,000 new cases and half a million deaths in 2020 [8]. Even though the incidence of esophageal adenocarcinoma is increasing, ESCC remains the most common histological type of cancer worldwide, with higher prevalence in East Asia and Japan [16,17]. The early diagnosis of ESCC has cure rates > 90%; however, early diagnosis remains a challenge that can be missed even on endoscopic examination [18]. Various diagnostic techniques, such as chromoendoscopy with iodine staining and narrow-band imaging (NBI), are helpful in detecting esophageal cancer at its early stages. While a diagnosis with only white light can be challenging, iodine staining can improve the sensitivity and specificity but can cause mucosal irritation, leading to retrosternal pain and discomfort to the patient [19,20,21,22]. NBI is another promising screening method for early esophageal cancer diagnosis [19,20]. Artificial intelligence can improve the sensitivity and specificity of diagnosis of esophageal cancer by improving the endoscopic and image diagnosis. Various retrospective and prospective studies have been conducted to study the role of different AI techniques in improving the diagnosis of esophageal cancer.

Retrospective studies included non-magnifying and magnifying images and real-time endoscopic videos of normal or early esophageal lesions to measure the diagnostic performance of AI models (23–24). One of the earliest retrospective studies conducted by Liu et al. with white light images used joint diagonalization principal component analysis (JDPCA), in which there are no approximation, iteration, or inverting procedures. Thus, JDPCA has low computational complexity and is suitable for the dimension reduction of gastrointestinal endoscopic images. A total of 400 conventional gastroscopy esophagus images were used from 131 patients, which showed an accuracy of 90.75%, with an area under the curve (AUC) of 0.9471 in detecting early esophageal cancer [23]. Another retrospective analysis was performed on ex-vivo volumetric laser endomicroscopy (VLE) images to analyze and compare various computer algorithms. Three novel clinically inspired algorithm features (“layering,” “signal intensity distribution,” and “layering and signal decay statistics”) were developed. When comparing the performance of these three clinical features and generic image analysis methods, “layering and signal decay statistics” showed better performance with sensitivity and specificity of 90% and 93%, respectively, along with an AUC of 0.81 compared to other methods tested [24]. 

Further retrospective analyses have been performed to analyze the role of different AI models, including supervised vector machines, convolutional neural networks on various diagnostic methods such as white light endoscopy, NBI, and real-time endoscopy videos. Cai et al. developed a computer-aided detection (CAD) using a deep neural network system (DNN) to detect early ESCC using 2428 esophageal conventional endoscopic white light images. DNN-CAD had sensitivity, specificity, and accuracy of 97.8%, 85.4%, and 91.45%, respectively, with a ROC of >96% when tested on 187 images from the validation dataset. Most importantly, the diagnostic ability of endoscopists improved significantly in sensitivity (74.2% vs. 89.2%), accuracy (81.7% vs. 91.1%), and negative predictive value (79.3% vs. 90.4%) after referring to the performance of DNN-CAD [25]. In another retrospective analysis performed by Horie et al., deep learning through convolutional neural networks were developed using 8428 training images of esophageal cancer, including conventional white light images and NBI. When tested on a set of 1118 test images, a CNN analyzed images in 27 s to accurately diagnose esophageal cancer with a sensitivity of 98%. It also showed an accuracy of 98% in differentiating superficial esophageal cancer from late-stage esophageal cancer, which can improve the prognosis of the patients and decrease the morbidity of more invasive procedures [26].

The definitive treatment of ESCC varies from endoscopic resection to surgery or chemoradiation depending on the level of invasion depth, so it is very important to determine it. In a study conducted in Japan, 1751 retrospectively collected training images of ESCC were used to develop an AI-diagnostic system of CNN using deep learning technique to detect the depth of invasion of ESCC. The AI-diagnostic system identified ESCC correctly in 95.5% of test images and estimated the invasion depth with a sensitivity of 84.1% and accuracy of 80.9% in about 6 s, which is higher than endoscopists [27]. Intrapapillary capillary loops (IPCLs) are microvessels seen visualized using magnification endoscopy. IPCLs are an endoscopic feature of early esophageal squamous cell neoplasia, and changes in their morphology are correlated with invasion depth. In this study, 7046 high-definition magnification endoscopies with NBI were used to train a CNN. As a result, CNN was able to identify abnormal from normal IPCLs patterns with an accuracy, sensitivity, and specificity of 93.7%, 89.3%, and 98%, respectively [28]. Based on various retrospective analyses, it was established that the use of AI in diagnosing esophageal cancer would prove beneficial. 

Many prospective analyses have also been performed to further assess the application of AI in the diagnosis of esophageal cancer. Struyvenberg et al. conducted a prospective study to detect Barrett’s neoplasia by CAD using a multi-frame approach. A total of 3060 VLE images were analyzed using a multi-frame analysis. Multi-frame analysis achieved a much higher AUC (median level = 0.91) than a single frame one (median level = 0.83). CAD was able to analyze multi-frame images in 3.9 s, which, traditionally, is a time-consuming and complex procedure due to the subtle gray shaded VLE images [29]. Thus, on the prospective study, as well, CAD proved beneficial for analyzing VLE images. Similarly, in another prospective study, a hybrid ResNet-UNet model CAD system was developed using five different independent endoscopic datasets to improve the identification of early neoplasm in patients with BE. When comparing the CAD system with general endoscopists, the study found higher sensitivity (93% vs. 72%), specificity (83% vs. 74%), and accuracy (88% vs. 73%) with the CAD system in the classification of images as containing neoplasm or non-dysplastic BE on dataset 5 (second external validation). CAD was also able to identify the optimal site to collect biopsy with higher accuracy of 97% and 92% of cases in datasets 4 and 5, respectively (dataset 4, 5 external validation sets, datasets 1, 2, and 3 were pre-training, training, and internal validation, respectively) [30].

With the promising results from retrospective and prospective studies conducted on endoscopic images, studies were designed to evaluate the role of AI for in vivo analysis to aid the diagnosis of Barrett’s during endoscopies. A prospective study developed and tested a CAD system to detect Barrett’s neoplasm during live endoscopic procedures. The CAD system predicted 25 of 33 neoplastic images and 96 of 111 non-dysplastic BE images correctly and thus had an image-based accuracy, sensitivity, and specificity of 84%, 76%, and 86%, respectively. Additionally, the CAD system predicted 9 of 10 neoplastic patients correctly, resulting in a sensitivity of 90%. So, this study showed high sensitivity to predict neoplastic lesions with the CAD system. However, it is in vivo single center, so further large multicenter trials are needed [31]. 

Shiroma et al. conducted a study to examine AI’s ability to detect superficial ESCC using esophagogastroduodenoscopy (EGD) videos. A CNN through deep learning was developed using 8428 EGD images of esophageal cancer. The AI system performance was evaluated using two validation sets of a total of 144 videos. The AI system correctly diagnosed 100% and 85% ESCC in the first and second validation sets, respectively. Whereas endoscopists detected only 45% of ESCC and their sensitivities improved significantly with AI real-time assistance compared to those without AI assistance (*p* < 0.05) [32]. In a retrospective study, a deep learning-based AI system was developed to detect early ESCC. Magnifying and non-magnifying endoscopy images of non-dysplastic, early ESCC, and advanced esophageal cancer lesions were used to train and validate the AI system. For non-magnifying images, AI diagnosis had a per-patient accuracy, sensitivity, specificity of 99.5%, 100%, and 99.5%, respectively, for white light imaging, and for magnified images, the per-patient accuracy, sensitivity, and specificity were 88.1%, 90.9%, and 85.0%, respectively. The accuracy of AI diagnosis was similar to experienced endoscopists; however, it was better than trainees [33].

Systematic reviews and meta-analyses of 21 and 19 studies, respectively, were conducted to test the CAD algorithm’s diagnostic accuracy to detect esophageal cancer using endoscopic images. It showed that the pooled AUC, sensitivity, specificity, and diagnostic odds ratio of CAD algorithms for the diagnosis of esophageal cancer for image-based analysis were 0.97 (95% CI: 0.95–0.99), 0.94 (95% CI: 0.89–0.96), 0.88 (95% CI: 0.76–0.94), and 108 (95% CI: 43–273), respectively. The pooled AUC, sensitivity, specificity, and diagnostic odds ratio of CAD algorithms for the diagnosis of esophageal cancer depth of invasion were 0.96 (95% CI: 0.86–0.99), 0.90 (95% CI: 0.88–0.92), 0.88 (95% CI: 0.83–0.91), and 138 (95% CI: 12–1569), respectively. There was no heterogeneity or publication bias on meta-regression [34]. Table 1 summarizes recent key studies assessing the role of AI in the diagnosis of esophageal cancer and pre-cancerous lesions using imaging [32,35,36,37,38,39,40,41,42,43,44,45,46].

The available literature provides strong evidence that the utilization of CAD in esophageal cancer can prove beneficial for early diagnosis, which remains crucial to prevent the significant morbidity and mortality of the patients [18]. While limitations such as external validation, clinical application, and the need for randomized control trials remain, the evidence so far supports the use of AI and therefore necessitates the need for larger controlled trials. 

## 3. Gastric Cancers

Gastric cancer is the sixth most common cancer worldwide by incidence with over 1 million new cases and the third leading cause of cancer-related death in 2020 [8]. The diagnosis of gastric cancer has transitioned from histology-only samples to precise molecular analysis of cancer. With the advent and use of endoscopy in diagnosing gastric cancer, the medical field is interested in earlier diagnosis in a non-invasive manner. The level of expertise required for endoscopic diagnosis of early gastric cancer remains high, and artificial intelligence can help with more accurate diagnosis and higher efficiency in image interpretation [47,48]. Here, we discuss the role of AI in diagnosing H. Pylori infection, a precursor of gastric cancer, and various methods of diagnosing gastric cancer with the help of machine learning methods.

### 3.1. Use of AI in Helicobacter Pylori Detection

Chronic, untreated H. Pylori infection is strongly associated with chronic gastritis, ulceration, mucosal atrophy, intestinal metaplasia, and gastric cancer [49]. On endoscopy, H. pylori infection is diagnosed by redness and swelling, which artificial intelligence can optimize. Various retrospective studies have been conducted to compare and develop a higher efficiency model of H. pylori diagnosis. Huang et al. pioneered the application of refined feature selection with neural network (RFSNN) developed using endoscopic images with histological features from 30 patients. It was then tested on 74 patients to predict H. pylori infection and related histological features. It showed sensitivity, specificity, and accuracy of 85.4%, 90.9%, and more than 80%, respectively [50]. A retrospective study developed a two-layered CNN model in which, first, CNN identified positive or negative H. pylori infection, and second, CNN classified the images according to the anatomical locations. The sensitivity, specificity, accuracy, and diagnostic time were 81.9%, 83.4%, 83.1%, and 198 s, respectively, for the first CNN and 88.9%, 87.4%, 87.7%, and 194 s, respectively, for the secondary CNN. Compared to the board-certified endoscopists, they were 85.2%, 89.3%, 88.9%, and 252.5 ± 92.3 min, respectively. The accuracy of the secondary CNN was significantly higher than all endoscopists, including relatively experienced and board-certified endoscopists (5.3%; 95% CI: 0.3–10.2), although they had comparable sensitivity and specificity [51]. Zheng et al. developed and used ResNet-50 based on endoscopic gastric images to diagnose H. pylori infection, which was confirmed with immunohistochemistry tests on biopsy samples or urea breath tests. The sensitivity, specificity, accuracy, and AUC were 81.4%, 90.1%, 84.5%, and 0.93, respectively for a single gastric image and 91.6%, 98.6%, 93.8%, and 0.97,respectively, for multiple gastric images [52]. These studies showed the high accuracy of CNN in diagnosing H. Pylori infection based on endoscopic imaging, and it was found to be comparable to the expert endoscopist.

A single-center prospective study compared the accuracy of the AI system with endoscopy images taken with white light imaging (WLI), blue laser imaging (BLI), and linked color imaging (LCL) in 105 H. pylori-positive patients. The AUC for WLI, BLI, and LCL were 0.66, 0.96, and 0.95, respectively (*p* < 0.01). Thus, this study showed a higher accuracy of H. pylori infection diagnosis with BLI and LCL with AI systems than WLI [53]. A systematic review and meta-analysis of eight studies were performed to evaluate AI accuracy in diagnosing H. pylori infection using endoscopic images. The pooled sensitivity, specificity, AUC, and diagnostic odds ratio to predict H. pylori infection were 87%, 86%, 0.92, and 40 (95% CI 15–112), respectively. The AI had a 40 times higher probability of predicting H. pylori infection than standard methods [54]. 

AI systems can be considered a valuable tool in the endoscopic diagnosis of H. Pylori infection based on available data from various studies. Although most of these studies lack external validation, promising results have been observed so far.

### 3.2. Use of AI in Gastric Cancer

Early diagnosis of gastric cancer remains prudent to provide less invasive and more successful treatments such as endoscopic submucosal dissection, which can be offered to patients with only intramucosal involvement [55]. AI can help by using endoscopy images for early diagnosis and thus better survival. A single-center observational study was conducted to test the efficacy of CAD for diagnosing early gastric cancer using magnifying endoscopy with narrow-band imaging. The CAD system was first pre-trained using cancerous and noncancerous images and then tested on 174 cancerous and noncancerous videos. The results showed a sensitivity, specificity, accuracy, PPV, NPV, and AUC of 87.4%, 82.8%, 85.1%, 83.5%, 86.7%, and 0.8684, respectively, for the CAD system. When comparing CAD against 11 expert endoscopists, the diagnostic performance of CAD was comparable to most expert endoscopists. Given the high sensitivity of CAD in diagnosing early gastric cancer, it can be helpful for endoscopists who are less experienced or lack endoscopic skills of ME-NBI. It can also be useful for experts with low diagnostic performance as diagnostic performance varies among experts [56]. Various CNN models have been developed to determine gastric cancer invasion depth, which can be used as a screening tool to determine the patient qualification for submucosal dissection. In another study, AI-based convolutional neural network computer-aided detection (CNN-CAD) was developed based on endoscopic images and then used to determine the invasion depth of gastric cancer. The AUC, sensitivity, specificity, and accuracy were 0.94, 76.47%, 95.56%, and 89.16%, respectively, for the CNN-CAD system.

Moreover, the CNN-CAD had an accuracy of 17.25% and a specificity of 32.21% higher than endoscopists [57]. Joo Cho et al. studied the application of a deep learning algorithm to determine the submucosal invasion of gastric cancer in endoscopic images. The mean AUC to discriminate submucosal invasion was 0.887 with external testing. Thus, deep learning algorithms may have a role in improving the prediction of submucosal invasion [58]. 

Ali et al. studied the application of AI on chromoendoscopy images to detect gastric abnormalities using endoscopic images. Chromoendoscopy is an advanced image-enhanced endoscopy technique that uses spraying dyes such as methylene blue to enhance gastric mucosa. This study uses a newer feature extraction method called Gabor-based gray-level co-occurrence matrix (G2LCM) for the computer-aided detection of chromoendoscopy abnormal frames. It is a hybrid approach of local and global texture descriptions. The G2LCM texture features and the support vector machine classifier were able to classify abnormal from normal frames with a sensitivity of 91%, a specificity of 82%, an accuracy of 87%, and an AUC of 0.91 [59]. In another study, CADx was trained with magnifying NBI and further with G2LCM-determined images from the cancerous blocks and compared to expert-identified areas. The CAD showed an accuracy of 96.3%, specificity of 95%, PPV of 98.3%, and sensitivity of 96.7%. This study showed that this CAD system could help diagnose early gastric cancer [60].

A systematic review and meta-analysis of 16 studies were performed to understand AI efficacy in endoscopic diagnosis of early gastric cancer. The use of AI in the endoscopic detection of early gastric cancer achieved an AUC of 0.96 (95%CI: 0.94–0.97), pooled sensitivity of 86% (95% CI: 77–92%), and a pooled specificity of 93% (95% CI: 89–96%). For AI-assisted depth distinction, the AUC, pooled sensitivity, and specificity were 0.82 (95% CI: 0.78–0.85), 72% (95% CI: 58–82%), and 79% (95% CI: 56–92%), respectively [61].

Most of the available literature currently focuses on AI applications in diagnosing gastric cancer rather than on the treatment response and prediction. Joo et al. constructed and studied the application of a one-dimensional convolution neural network model (DeepIC50), which showed accuracy in pan-cancer cell line prediction. This was applied to approved treatments of trastuzumab and ramucirumab, which showed promising predictions for drug responsiveness, which can be helpful in the development of newer medication [62].

While many studies have been conducted independently, there is a need for larger prospective trials studying the application of AI in the entirety of gastric cancer diagnosis and treatment to better assess its efficacy and application in clinical practice. Table 2 summarizes key studies assessing the role of AI in the diagnosis of Gastric cancer by imaging [23,63,64,65,66,67,68,69,70].

## 4. Colorectal Cancer

Colorectal cancer (CRC) is the fourth most common cancer diagnosed in the United States [71]. Its incidence has been falling steadily, mostly due to screening using colonoscopy and treatment of polyps that can effectively prevent the development of colon cancer [71,72]. The use of AI is increasingly studied to improve polyp and cancer detection in the colon [73].

### 4.1. AI and Colon Polyp Detection

A recent systematic review and meta-analysis reported a pooled sensitivity and specificity of 92% and 93%, respectively, with 92% accuracy using still and video images from colonoscopy in over 17,400 patients. When using video frames or images, the pooled sensitivity and specificity were higher (92% and 89%, respectively) compared to studies that used still images alone, reporting a pooled sensitivity and specificity of 84% and 87%, respectively, for AI in the detection of colon polyps. Most of the studies were retrospective in design [74]. The types of AI used ranged from SVM, ANN, and CNN to several modifications of deep learning methods. A meta-analysis of seven randomized controlled trials (RCTs) showed a significant increase in the rate of polyp detection when AI was used with colonoscopy compared to colonoscopy alone, with an odds ratio of 1.75 (95% CI: 1.56–1.96, *p* < 0.001). All studies had a higher polyp detection rate in the AI group than the standard colonoscopy alone group [74]. Recent studies have also shown considerable promise in the use of AI, especially CNN-based systems in colon capsule endoscopy, to improve rates of colon polyp detection [75,76]. Laiz et al. developed a CNN model to detect polyps of all sizes and morphologies using capsule endoscopy images and reported specificity of over 90% for small to large size polyps as well as pedunculated or sessile polyps [76].

### 4.2. AI and Colon Polyp Characterization

A pooled analysis of 20 studies that used AI to predict the histology of a polyp detected on colonoscopy revealed a sensitivity and specificity of 94% and 87% with 91% accuracy in detecting an adenomatous polyp. SVM was the most common AI in these studies, followed by DNN, CNN, and other DL methods [74]. A meta-analysis of seven RCTs also showed an increased detection rate of adenomas when AI was combined with colonoscopy compared to colonoscopy alone with an OR 1.53 (95%CI: 1.32–1.77, *p* < 0.001). The absolute improvement in adenoma detection ranged from 6% to 15.2% for AI compared to colonoscopy alone in the studies. All RCTs utilized ANN- or CNN-based AI systems using video streams from colonoscopy [74]. Table 3 summarizes select studies assessing role of AI in diagnosis/characterization of polyps and colon cancer by imaging [77,78,79,80,81,82,83,84,85,86,87,88,89,90,91,92,93,94,95,96,97,98,99].

### 4.3. AI and Colorectal Cancer

Lymph nodal metastases in colon cancer confer a lower survival and prognosis than patients with no nodal metastases in early-stage colon cancer, with 5-year survival dropping from over 90% to 72% in the presence of nodal metastases in early-stage colon cancer [71]. Kudo et al. studied an ANN-based system in over 3000 patients with T1 colorectal cancers and found a significantly higher nodal metastasis rate identified (AUC 0.83) compared to standard guidelines alone (AUC 0.73, *p* < 0.001). Analyzing patients who underwent the endoscopic resection of T1 tumors, the LN metastases rate was higher for the AI system compared to guidelines (AUC 0.84 vs. AUC 0.77, *p* = 0.005), signifying a potential role for AI in detecting patients who may need further nodal sampling after the endoscopic resection of early-stage colon cancer [100]. Ichimasa et al. studied AI in 590 resected patients with T1 colorectal cancer and found that AI was 100% sensitive for LN metastases detection with a specificity and accuracy over 20% points higher than US clinical guidelines alone [101].

Overall, AI has shown considerable promise to be an excellent addition to a gastroenterologist’s armamentarium to not only detect small polyps with higher accuracy but also to be able to differentiate adenomas from other histologies and identify patients with early-stage T1 colon cancer who may benefit from or may be able to avoid additional nodal surgery.

## 5. Pancreatic Cancer

Pancreatic cancer is an aggressive cancer with a dismal 5-year overall survival of <10%. The incidence is rising globally, and it has the highest mortality rate amongst all major cancers [102]. Surgical resection at an early stage remains the only chance of cure for patients, hence signifying the importance of early detection of these malignancies. Artificial intelligence has been studied to augment the current diagnostic armamentarium to help identify pancreatic lesions and differentiate benign from malignant disease [103]. Here, we will discuss the evidence so far about the role of AI in pancreatic cancers.

### 5.1. AI and Radiologic Diagnosis of Pancreatic Cancer

Liu et al. using a faster region-based CNN model reported an AUC of 0.96 for the recognition of pancreatic cancer based on contrast-enhanced CT images. In contrast, Li et al. reported an accuracy of only 72.8% of differentiating various pancreatic cysts based on CT images using densely connected convolutional networks [104,105]. Using the ML approach, Chu et al. reported a sensitivity of 100%, a specificity of 98.5%, and a 99% accuracy [106]. Wei et al. studied the SVM model and reported a much lower sensitivity and specificity of 66.7 and 81.8%, respectively [107]. Li et al. studied a method combining SVM and random forest technology using PET/CT images and showed excellent sensitivity, specificity, and accuracy of 95%, 97.5%, and 96%, respectively [108]. All these studies had small sample sizes of a few hundred patients.

AI application has been challenging in MR images for the recognition of pancreatic neoplasms. Kaisiss et al., using the ML model, reported an AUC of 0.93 with the sensitivity of 84% and specificity of 92% for the recognition of pancreatic cancers. In comparison, Corral et al. reported an efficacy of 78% using a DL model with a sensitivity of 92% and a specificity of 52% for recognizing malignant IPMNs [109,110].

### 5.2. AI and Endoscopic Diagnosis of Pancreatic Cancer

Several studies have reported excellent accuracy of detection of pancreatic cancer from the normal pancreas. Ozkan et al. studied ANN and reported an accuracy of 91.66% in patients over 60 years old, while Mayra et al. and Tonozuka et al. studied CNN with a reported ROC AUC of 0.94 and 0.957, respectively, to identify pancreatic cancer from the noncancerous pancreas [111,112,113]. All studies still used EUS images. Saftoiu et al. used video images collected prospectively and fed in an ANN model with a reported accuracy of 89.7% to differentiate malignant from benign patterns [114].

The presence of chronic pancreatitis complicates the diagnosis of pancreatic cancer, where standard EUS has low specificity, and cytology remains the mainstay of diagnosis. AI has been shown to improve the diagnostic capability of EUS to differentiate cancer from chronic pancreatitis compared to cytology. Zhang et al. showed an accuracy of 94% with 93% specificity to differentiate cancer from chronic pancreatitis using a support vector machine model of AI in a retrospective analysis of 388 patients using still EUS images [115]. Three prospective studies were conducted by Saftiou et al. using video images of EUS fed in an ANN model also reported a high accuracy (90%) with sensitivities 88–95% and specificities 83–94%, much higher than traditional EUS alone [114,116,117].

Kuwahara et al. showed that the CNN model of AI had a 94% accuracy in differentiating malignant from benign IPMNs. They also reported very high sensitivity, specificity, PPV, and NPV of 96%, 93%, 92%, and 96%, respectively. However, this study was a small retrospective analysis of still EUS images from 50 patients (23 malignant and 27 benign IPMNs) [118].

### 5.3. AI and microRNA (miRNA) for Diagnosis of Pancreatic Cancer

Non-invasive biomarkers such as CA19-9 are non-specific in the diagnosis of pancreatic cancer. MicroRNA expressions are found to be unique in different gastrointestinal cancers, and their small size, stability, and easy detection in serum, make them potentially attractive for the early diagnosis of pancreatic cancer [119,120]. Yan et al. studied 100 pancreatic cancer patients and 150 controls to identify 13 miRNA expressions unique to pancreatic cancer with sensitivity, specificity, and accuracy of 80%, 97.6%, and 91.6%, respectively, and it was better than conventional biomarkers (CEA and CA19-9) [121]. Savareh et al. combined five unique miRNAs with an AI model consisting of an ANN with Particle Swarm Optimization (PSO) and Neighborhood Component Analysis (NCA) and reported a sensitivity, specificity, and accuracy of 93%, 92%, and 93%, respectively [122]. Sinkala et al. showed that combining AI with mRNA, miRNA, and DNA methylation patterns can recognize two distinct molecular subtypes of pancreatic cancer (one aggressive and one not so aggressive) that may have therapeutic implications [123].

Overall, AI has shown considerable promise in the early detection of pancreatic malignancies and may become a useful adjunct to interventional gastroenterologists in the near future. Studies with CT scans and MRI showed a range of sensitivities and specificities, while a custom model of AI with PET/CT images showed promise. Endoscopic studies using AI in EUS imaging analysis showed higher sensitivities and specificities than traditional EUS to recognize pancreatic malignancies, and AI combined with miRNA also shows promise. If successful, they may help establish a diagnosis of pancreatic cancer in a non-invasive manner without cytological confirmation.

## 6. Hepatocellular Cancer

Hepatocellular cancer (HCC) is a common hepatic tumor that can be diagnosed with specific radiological criteria without the need for tissue biopsy in the appropriate patient population [124]. Less than half of the patients present with the local disease that is potentially curable with a 5-year OS of 35%, while patients with distant metastases have a dismal 5-year OS survival of <3% [79]. AI has the potential to augment the accuracy of diagnosis of early HCC and help increase the likelihood of early diagnosis and treatment. Here, we discuss the evidence supporting the role of AI in the diagnosis of HCC.

### 6.1. AI and Ultrasound Diagnosis of HCC

Virmani et al. used the SVM technique to obtain a classification accuracy of 88.8% [125]. Wu et al. used a fused feature model comprising of k-NN, fuzzy-NN, PNN, and SVM to obtain a diagnostic accuracy of 96.6% to identify HCC, while Lee et al. and Bharti et al. used multiple AI techniques (k-NN, fuzzy-NN, PNN, SVM) and combined them using CNN-DL techniques (ensemble model) to obtain a classification accuracy of 95.7% and 96.6%, respectively, to differentiate HCC from the normal and cirrhotic liver [126,127,128]. Schmauch et al. used a DL algorithm on 177 patients to obtain the ROC-AUC of 0.931 for the characterization of HCC amongst focal liver lesions [129].

### 6.2. AI and CT-Scan Diagnosis of HCC

Cao et al. utilized a deep neural network (DNN)—automated multiphase convolutional dense network (MP-CDN) in 375 patients with 517 lesions to classify liver lesions into four groups (A-HCC, B-liver metastases, C-benign non-inflammatory lesions such as cysts, adenomas, and D-liver abscesses). They reported that the AUCs for differentiating each category from the others were 0.92, 0.99, 0.88, and 0.96, respectively, for HCC, metastases, benign non-inflammatory lesions, and abscesses [130]. Yasaka et al. applied a CNN model to CT-scan images of 100 patients with liver lesions and divided them into five groups (A-classic HCC, B-non-HCC tumors, C-indeterminate lesions, or benign solid masses, D-hemangiomas, E-cysts). They reported an overall accuracy of 84% with an ROC curve of 0.92 to differentiate A-B from C-E lesions [131]. 

### 6.3. AI and MRI Diagnosis of HCC

Hamm et al. applied the CNN model to multiphasic MRI images in 494 hepatic lesions and compared the performance of the AI model to radiologists. The CNN model showed a sensitivity and specificity of 92% and 98%, respectively, compared to 82.5% sensitivity and 96.5% specificity for radiologists reading the same cases. The overall accuracy of AI was 92%, with a false positive rate of 1.6% and an ROC of 0.992 [132]. Oestmann et al. also studied a CNN model in 118 patients with 150 pathologically confirmed liver lesions to differentiate HCC from non-HCC lesions. The model showed a sensitivity and specificity of 92.7% and 82%, respectively, to identify HCC with an overall accuracy of 87.3% and ROC of 0.912 [133].

Overall, AI has shown considerable progress in application with US, CT-scans, and MRIs to increase HCC diagnosis accuracy and early detection, with some studies showing better sensitivities and specificities than radiologists. AI has the potential to become an excellent adjunct to radiology techniques in diagnosing HCC in the future.

## 7. AI in Histopathologic Diagnosis of GI Malignancies

Several studies have explored the role of AI in assisting with pathological diagnosis of GI cancers. Of all AI methodologies, CNN has been the most widely studied with regards to applications in the pathological diagnosis of cancer. If well built, it has the potential to ease the pathologist’s workload and increase the efficiency of pathological diagnosis [9,134]. While Sharma et al. reported an accuracy of 0.699 for the classification of cancer with DL methodology applied to digital images of H&E-stained whole slide, Iizuka et al. reported AUCs of 0.924 and 0.982 for the histological classification of gastric adenocarcinoma and colon adenocarcinoma, respectively, using DL methodology [135,136]. Kuntz et al. reported a systematic review of 16 studies using CNN in making histologic diagnosis or assessing the prognosis of CRC and GC. All studies assessing molecular characteristics were conducted in CRC, and no study on the use of AI in assessing the molecular characteristics of gastric or esophageal cancer was found. There was a wide range of sensitivities and specificities of different studies with sensitivities ranging from 52 to 100% and specificities from 57 to 100%. The performance of AI was as good as or better than pathologists. However, all studies were retrospective and there was considerable variability in the types of CNN methodologies applied, with 14 different CNN techniques in 16 studies [9]. Momemi-Boroujeni et al. studied multilayer perception neural network to recognize benign, malignant, and atypical pancreatic lesions. Although AI was 100% accurate at differentiating benign from malignant pathology, the accuracy fell to 77% for atypia [137]. Due to the large amount of variability in reported data, AI is not yet ready for real time application in pathology practice to diagnose GI malignancies, but the ongoing work is extremely promising [138]. Overall, there is a need to identify an AI model for pathological diagnosis that is generalizable on a large scale and can be commercially developed for applicability in clinical practice.

## 8. Conclusions

This review outlined the current published literature on the AI application in gastrointestinal, pancreatic, and hepatocellular cancers. AI is considered an instrumental tool in changing the future of healthcare, especially oncology. AI may become a useful tool in screening, diagnosing, and treating various cancers by accurately analyzing diagnostic clinical images, identifying therapeutic targets, and processing large datasets. Although the diagnostic accuracy of AI systems has markedly increased, it still needs collaboration with physicians. Robertson et al. proposed a five-step process that they foresee can help integrate AI with clinical practice, namely, Quality improvement, Productivity improvement, and Performance improvement, followed by Evaluation (step 4) where AI replaces human analysis and the results can be reviewed by the gastroenterologist before being reported to the final step 5, Diagnostic, where AI may replace the diagnostician for simple pathologic results releasing them to the patients without review [75]. However, before we can follow these steps, there is a need to identify one or two methodologies from numerous choices that can be developed, generalized, and commercialized. Most of the data on AI use are not prospective, hence large and multicenter clinical trials are needed to further validate this system in real-time clinical settings. If successful, the AI-assisted systems have the potential to become a vital tool for the management of these cancer patients.

## Figures and Tables

**Table 1:** Studies showing the application of AI in the early detection of esophageal cancer by imaging. AUC: Area under the receiver operating characteristic curve, BLI: Blue-laser imaging, BE: Barrett’s esophagus, CAD: Computer-aided detection, CNN: Convolutional Neural Networks, DNN-CAD: Deep neural network computer-aided network, HRME: High-resolution micro endoscopy, MICCAI: Medical Image Computing and Computer-Assisted Intervention, NBI: Narrow-Band imaging, SVM: Support vector machine, VLE: Volumetric laser endomicroscopy, WLI: White light images.

Author, Year, Reference	Dataset (Images Count and Lesions Type)	AI System	Modality	Results
Shin 2015 [35]	375 images (esophageal squamous cell cancer)	Two-class linear discriminant analysis	HRME	Sensitivity 84%, specificity 95%, and AUC 0.95
Quang 2016 [36]	375 images (esophageal squamous cell cancer)	Fully automated real-time analysis algorithm	HRME	Sensitivity 95%, specificity 91%, and AUC 0.937
Van der Sommen 2016 [37]	100 images (60 early BE neoplasia and 40 BE)	SVM	WLI	Per image-sensitivity 83% and specificity 83%. For per patient sensitivity 86% and specificity 87%.
Swager 2017 [24]	60 images (30 early BE neoplasia and 30 BE)	SVM	VLE	Sensitivity 90%, specificity 93%, and AUC 0.95
Mendel 2017 [38]	100 (50 BE and 50 esophageal adenocarcinoma)	CNN	WLI	Sensitivity 94% and specificity 88%
Cai 2019 [25]	2615 images (early esophageal squamous cell cancer)	DNN-CAD	WLI	Sensitivity 97.8%, specificity 85.4 %, and accuracy 91.4%
Horie 2019 [26]	9546 images (esophageal cancer)	CNN-SSD (single shot multibox detector)	WLI and NBI	Per image Sensitivity 72% (WLI) and 86% (NBI). Per case Sensitivity 79% (WLI) and 89% (NBI).
Ebigbo 2019 [39]	248 images. Two databases. [(a)- Augsburg dataset-148. (b) MICCAI dataset-100]	CNN-ResNet (residual net)	WLI and NBI	Augsburg database—WLE sensitivity 97% and specificity 88% and NBI sensitivity 94% and specificity 80% MICCAI database—sensitivity 92% and specificity 100%
Everson 2019 [28]	7046 images (Intrapapillary capillary loop patterns in early esophageal squamous cell cancer)	CNN	Magnified NBI	Sensitivity 89.3%, specificity 98%, and accuracy 93.7%.
Zhao 2019 [40]	1350 images (early esophageal squamous cell cancer)	Double labeling fully convolutional network (FCN)	Magnifying endoscopy with NBI	Diagnostic accuracy at the lesion level 89.2% and at the pixel level 93.0%.
Nakagawa 2019 [41]	15252 images (early esophageal squamous cell cancer)	CNN	Magnified and non-magnified WLI, NBI, and BLI	Sensitivity 90.1%, specificity 95.8%, and accuracy 91%
Guo 2019 [42]	6473 images and 47 videos (early esophageal squamous cell cancer)	CNN-SegNet	Non-magnified and magnified NBI	Per image sensitivity 98.04%, specificity 95.03%, and AUC 0.989 Per frame sensitivity 91.5% and specificity 99.9%
Hashimoto 2020 [43]	1832 images (916 early BE neoplasia and 916 BE)	CNN	WLI and NBI	WLI sensitivity 98.6% and specificity 88.8%. NBI sensitivity 92.4% and specificity 99.2%
Ohmori 2020 [44]	23289 (superficial early esophageal squamous cell cancer)	CNN	Non-magnified WLI, NBI, and BLI. Magnified NBI and BLI	Non-magnified NBI/BLI—sensitivity 100%, specificity 63%, and accuracy 77%. Non-magnified WLI—sensitivity 90%, specificity 76%, and accuracy 81%. Magnified NBI—sensitivity 98%, specificity 56%, and accuracy 77%.
Tokai 2020 [27]	1751 (superficial early esophageal squamous cell cancer)	CNN	WLI and NBI	Sensitivity 84.1%, specificity 73.3%, and accuracy 80.9%
Li 2021 [45]	2167 images (early esophageal squamous cell cancer)	CAD	WLI and NBI	CAD-NBI sensitivity 91%, specificity 96.7%, and accuracy 94.3%. CAD-WLI sensitivity 98.5%, specificity 83.1%, and accuracy 89.5%
Shiroma 2021 [32]	8428 and 80 videos (T1 esophageal squamous cell cancer)	CNN	WLI and NBI	WLI sensitivity 75%, specificity 30% NBI sensitivity 55%, specificity 80%
Ebigbo 2021 [46]	230 WLI images (108 T1a, 122 T1b stage)	ANN	WLI	Sensitivity 77%, Specificity 64%, Diagnostic accuracy 71% to differentiate T1a from T1b lesions. Not significantly different from clinical experts

**Table 2:** Studies showing application of AI in early detection of gastric cancer by imaging. AUC: Area under the curve, JDPCA: Joint diagonalization principal component analysis, BLI: Blue-laser imaging, CNN: Convolutional neural networks, CNN-CAD: Convolutional neural network computer aided-diagnosis, G2LCM: Gabor-based gray-level co-occurrence matrix, GLCM: gray-level co-occurrence matrix, LCI: linked color imaging, NBI: Narrow-band imaging, RNN: recurrent neural networks, SVM: Support vector machine, WLI: white light imaging.

Author, Year, Reference	Dataset (Images Count and Lesions Type)	AI System	Modality	Results
Miyaki 2015 [63]	587 cut out images, early gastric cancer	SVM	Magnifying endoscopy-BLI	SVM output 0.846 ± 0.220 for cancerous lesions and 0.219 ± 0.277 for surrounding tissues
Liu 2016 [23]	400 images, early gastric cancer	JDPCA	WLI	AUC—0.9532, accuracy—90.75%
Shichijo 2017 [39]	32,208 images (CNN 1) and images classified based on 8 anatomic locations (CNN2), Helicobacter pylori infection	Deep CNN	WLI	CNN 1—Sensitivity 81.9%, specificity 83.4%, and accuracy 83.1% CNN2—Sensitivity 88.9%, specificity 87.4%, and accuracy 87.7%
Ali 2018 [46]	176 images, abnormal gastric mucosa including metaplasia and dysplasia	G2LCM	Chromoendoscopy	Sensitivity 91%, specificity 82%, accuracy 87%, and AUC 0.91
Hirasawa 2018 [64]	13584 endoscopic images, gastric cancer	CNNS bases single shot Multibox Detector	WLE, NBI and chromoendoscopy	Sensitivity 92.2%
Kanesaka 2018 [47]	126 images, early gastric cancer	GLCM features, SVM	Magnifying endoscopy NBI	Sensitivity 96.7%, specificity 95%. and accuracy 96.3%
Liu 2018 [65]	1120 Magnifying endoscopy NBI images, early gastric cancer	Deep CNN	Magnifying endoscopy NBI	Top Sensitivity 96.7%, specificity 95%. and accuracy 98.5%
Horiuchi 2019 [66]	2828 images (1643 early gastric cancer, 1185 gastritis, early gastric cancer	CNN	Magnifying endoscopy NBI	Sensitivity 95.4%, specificity 71%, and accuracy 85.3%
Zhu 2019 [45]	993 images, Invasive depth of gastric cancer invasion	CNN-CAD system	WLI	Sensitivity 76.47%, specificity 95.56%, accuracy 89.1%, and AUC 0.98
Guimaraes 2020 [67]	270 images, gastric precancerous condition such as atrophic gastritis	Deep learning, CNN	WLI	AUC 0.98, sensitivity 93%
Yasuda 2020 [68]	525 images, H. pylori infection	SVM	LCI	Sensitivity 90.4%, specificity 85.7%, and accuracy 87.6%
Wu 2021 [69]	1050 patients, early gastric cancer	ENDOANGEL- deep CNN based system	WLI	Per lesion, sensitivity 100%, specificity 84.3%, and accuracy 84.7%
Xia 2021 [70]	1,023,955 images, gastric lesion	Faster region-based convolutional neural network	Magnetically controlled capsule endoscopy	Sensitivity 96.2%, specificity 76.2%, and accuracy 77.1%

**Table 3:** Studies on AI in colorectal polyp detection and characterization using imaging. AUC: Area under the receiver operating characteristic curve, CNN: Convolutional neural network, CAD: Computer-aided diagnosis, CADe: Real-time computer-aided detection, DNN: Deep neural network, EC-CAD: Computer-aided diagnostic system for endocytoscopic imaging, NBI: Narrow-band imaging, SVM: Support vector machine, WLI: White light imaging, WM-DOVA: Window Median of Valleys Accumulation.

Author, Year, and Reference	Dataset	AI System	Modality	Results
Tischendorf 2010 [77]	209 polyps, colorectal polyps	Region growing algorithm	Magnifying NBI	Sensitivity 90% and specificity 70%.
Takemura 2012 [78]	371 colorectal lesions	SVM	Magnification chromoendoscopy	Sensitivity 97.8%, specificity 97.9%, and accuracy 97.8%
Mori 2015 [79]	176 colorectal lesions	SVM, EC-CAD	WLI, endocytoscopy	Sensitivity 92.0%, specificity 79.5%, and accuracy 89.2%
Fernandez 2016 [80]	31 colorectal polyps from 24 videos	WM-DOVA energy maps	WLI	Sensitivity 70.4% and specificity 72.4%
Kominami 2016 [81]	118 colorectal lesions	Real-time CAD and SVM	Magnifying NBI	Sensitivity 93.0%, specificity 93.3%, and accuracy 93.2%
Park and Sargent 2016 [82]	11802 images patches	CNN	WLI and NBI	Sensitivity 86% and specificity 85%
Tamai 2017 [83]	121 colorectal lesions	CAD	Magnifying NBI	Sensitivity 83.9%, specificity 82.6% ,and accuracy 82.8%
Zhang 2017 [84]	215 colorectal polyps	CNN	WLI and NBI	Precision 87.3 and accuracy 85.9%
Misawa 2018 [85]	155 polyps from 73 colonoscopy videos	CNN	WLI	Per frame: Sensitivity 90%, specificity 63.3%, and accuracy 76.5%
Mori 2018 [86]	466 diminutive colorectal polyps from 325 patient	CAD, SVM	NBI	Pathologic prediction rate 98.1%
Urban 2018 [87]	8,641 images from screening colonoscopy containing 4088 colorectal polyp detection	CNN	WLI	Accuracy 96.4%, AUC 0.991
Bryne 2019 [88]	125 diminutive colorectal polyp videos	Deep CNN	NBI	Sensitivity 98%, specificity 83%, and accuracy 94%
Figueiredo 2019 [89]	1680 frames with polyps and 1360 frames without polyps from 42 patients	SVM binary classifiers	WLI	Sensitivity 99.7%, specificity 84.9%, and accuracy 91.1%
Horiuchi 2019 [90]	429 diminutive colorectal polyps (258 rectosigmoid and 171 non-rectosigmoid polyps)	Color intensity analysis software	Autofluorescence imaging	Sensitivity 80.0 %, specificity 95.3%, and accuracy 91.5%
Ito 2019 [91]	190 images of colon lesions, stage 1b colon cancer	CNN	WLI	Sensitivity 67.5%, specificity 89%, accuracy 81.2%, and AUC 0.871
Wang 2019 [92]	1058 patients (536 randomized to standard colonoscopy and 522 to colonoscopy with computer aided diagnosis)	CNN	WLI	Adenoma detection rate 29.1% for standard colonoscopy and 20.3% for colonoscopy with computer-aided diagnosis group
Jin 2020 [93]	300 images of colorectal polyps (180 adenomatous polyps and 120 hyperplastic polyps)	CNN	NBI	Sensitivity 83.3 %, specificity 91.7%, and accuracy 86.7%
Kudo 2020 [94]	2000 images, colorectal polyps	Endocytoscopy with NBI and methylene blue staining modes	EndoBRAIN	Sensitivity 96.9%, specificity 100%, and accuracy 98%
Nakajima 2020 [95]	78 images, Colorectal cancer with deep submucosal invasion	CAD	Non magnified WLI	Sensitivity 81%, specificity 87%, and accuracy 84%
Ozawa 2020 [96]	1172 colorectal polyp images from 309 polyps.	CNN	NBI	Trained CNN detection—Sensitivity 92% and positive predictive value 86%. Colorectal polyp characterization with NBI—sensitivity 97% and positive predictive value 98%.
Repici 2020 [97]	685 patients who underwent screening colonoscopy	CADe	WLI	Adenoma detection rate for CADe (54.8%) higher than standard colonoscopy (40.4%) with relative risk 1.30, 95% Cl: 1.14–1.45
Lai 2021 [98]	16 patients, colorectal polyps	DNN	WLI and NBI	Sensitivity 100%, specificity 100%, and accuracy 74–95%
Luo 2021 [99]	150 patients who underwent screening colonoscopy	CNN	WLI	Polyp detection rate for AI-assisted colonoscopy group (38.7%) higher than standard colonoscopy group (34.0%), *p* <0.001.

## References

[1] Russell S., Norvig P. (2009). Artificial Intelligence: A Modern Approach.

[2] Shalev-Shwartz S., Ben-David S. (2014). Understanding Machine Learning: From Theory to Algorithms.

[3] Mitsala A., Tsalikidis C., Pitiakoudis M., Simopoulos C., Tsaroucha A.K. (2021). Artificial Intelligence in Colorectal Cancer Screening, Diagnosis and Treatment. A New Era. Curr. Oncol..

[4] Deo R.C. (2015). Machine Learning in Medicine. Circulation.

[5] LeCun Y., Bengio Y., Hinton G. (2015). Deep learning. Nature.

[6] Kudou M., Kosuga T., Otsuji E. (2020). Artificial intelligence in gastrointestinal cancer: Recent advances and future perspectives. Artif. Intell. Gastroenterol..

[7] Ruffle J.K., Farmer A.D., Aziz Q. (2019). Artificial Intelligence-Assisted Gastroenterology- Promises and Pitfalls. Am. J. Gastroenterol..

[8] Bray F., Ferlay J., Soerjomataram I., Siegel R.L., Torre L.A., Jemal A. (2018). Global cancer statistics 2018: GLOBOCAN estimates of incidence and mortality worldwide for 36 cancers in 185 countries. CA A Cancer J. Clin..

[9] Kuntz S., Krieghoff-Henning E., Kather J.N., Jutzi T., Hohn J., Kiehl L., Hekler A., Alwers E., von Kalle C., Frohling S. (2021). Gastrointestinal cancer classification and prognostication from histology using deep learning: Systematic review. Eur. J. Cancer.

[10] Suzuki H., Yoshitaka T., Yoshio T., Tada T. (2021). Artificial intelligence for cancer detection of the upper gastrointestinal tract. Dig. Endosc..

[11] Huynh J.C., Schwab E., Ji J., Kim E., Joseph A., Hendifar A., Cho M., Gong J. (2020). Recent Advances in Targeted Therapies for Advanced Gastrointestinal Malignancies. Cancers.

[12] Que S.J., Chen Q.Y., Qing Z., Liu Z.Y., Wang J.B., Lin J.X., Lu J., Cao L.L., Lin M., Tu R.H. (2019). Application of preoperative artificial neural network based on blood biomarkers and clinicopathological parameters for predicting long-term survival of patients with gastric cancer. World J. Gastroenterol..

[13] Le Berre C., Sandborn W.J., Aridhi S., Devignes M.-D., Fournier L., Smaïl-Tabbone M., Danese S., Peyrin-Biroulet L. (2020). Application of Artificial Intelligence to Gastroenterology and Hepatology. Gastroenterology.

[14] He Y.S., Su J.R., Li Z., Zuo X.L., Li Y.Q. (2019). Application of artificial intelligence in gastrointestinal endoscopy. J. Dig. Dis..

[15] Lech G., Słotwiński R., Słodkowski M., Krasnodębski I.W. (2016). Colorectal cancer tumour markers and biomarkers: Recent therapeutic advances. World J. Gastroenterol..

[16] Enzinger P.C., Mayer R.J. (2003). Esophageal cancer. N. Engl. J. Med..

[17] Kuwano H., Nishimura Y., Oyama T., Kato H., Kitagawa Y., Kusano M., Shimada H., Takiuchi H., Toh Y., Doki Y. (2015). Guidelines for Diagnosis and Treatment of Carcinoma of the Esophagus April 2012 edited by the Japan Esophageal Society. Esophagus.

[18] Naveed M., Kubiliun N. (2018). Endoscopic Treatment of Early-Stage Esophageal Cancer. Curr. Oncol. Rep..

[19] Kuraoka K., Hoshino E., Tsuchida T., Fujisaki J., Takahashi H., Fujita R. (2009). Early esophageal cancer can be detected by screening endoscopy assisted with narrow-band imaging (NBI). Hepatogastroenterology.

[20] Nagami Y., Tominaga K., Machida H., Nakatani M., Kameda N., Sugimori S., Okazaki H., Tanigawa T., Yamagami H., Kubo N. (2014). Usefulness of non-magnifying narrow-band imaging in screening of early esophageal squamous cell carcinoma: A prospective comparative study using propensity score matching. Am. J. Gastroenterol..

[21] Kondo H., Fukuda H., Ono H., Gotoda T., Saito D., Takahiro K., Shirao K., Yamaguchi H., Yoshida S. (2001). Sodium thiosulfate solution spray for relief of irritation caused by Lugol’s stain in chromoendoscopy. Gastrointest. Endosc..

[22] Menon S., Trudgill N. (2014). How commonly is upper gastrointestinal cancer missed at endoscopy? A meta-analysis. Endosc. Int. Open.

[23] Liu D.-Y., Gan T., Rao N.-N., Xing Y.-W., Zheng J., Li S., Luo C.-S., Zhou Z.-J., Wan Y.-L. (2016). Identification of lesion images from gastrointestinal endoscope based on feature extraction of combinational methods with and without learning process. Med. Image Anal..

[24] Swager A.F., van der Sommen F., Klomp S.R., Zinger S., Meijer S.L., Schoon E.J., Bergman J., de With P.H., Curvers W.L. (2017). Computer-aided detection of early Barrett’s neoplasia using volumetric laser endomicroscopy. Gastrointest. Endosc..

[25] Cai S.L., Li B., Tan W.M., Niu X.J., Yu H.H., Yao L.Q., Zhou P.H., Yan B., Zhong Y.S. (2019). Using a deep learning system in endoscopy for screening of early esophageal squamous cell carcinoma (with video). Gastrointest. Endosc..

[26] Horie Y., Yoshio T., Aoyama K., Yoshimizu S., Horiuchi Y., Ishiyama A., Hirasawa T., Tsuchida T., Ozawa T., Ishihara S. (2019). Diagnostic outcomes of esophageal cancer by artificial intelligence using convolutional neural networks. Gastrointest. Endosc..

[27] Tokai Y., Yoshio T., Aoyama K., Horie Y., Yoshimizu S., Horiuchi Y., Ishiyama A., Tsuchida T., Hirasawa T., Sakakibara Y. (2020). Application of artificial intelligence using convolutional neural networks in determining the invasion depth of esophageal squamous cell carcinoma. Esophagus.

[28] Everson M., Herrera L., Li W., Luengo I.M., Ahmad O., Banks M., Magee C., Alzoubaidi D., Hsu H.M., Graham D. (2019). Artificial intelligence for the real-time classification of intrapapillary capillary loop patterns in the endoscopic diagnosis of early oesophageal squamous cell carcinoma: A proof-of-concept study. United Eur. Gastroenterol. J..

[29] Struyvenberg M.R., van der Sommen F., Swager A.F., de Groof A.J., Rikos A., Schoon E.J., Bergman J.J., de With P.H.N., Curvers W.L. (2020). Improved Barrett’s neoplasia detection using computer-assisted multiframe analysis of volumetric laser endomicroscopy. Dis. Esophagus.

[30] de Groof A.J., Struyvenberg M.R., van der Putten J., van der Sommen F., Fockens K.N., Curvers W.L., Zinger S., Pouw R.E., Coron E., Baldaque-Silva F. (2020). Deep-Learning System Detects Neoplasia in Patients with Barrett’s Esophagus With Higher Accuracy Than Endoscopists in a Multistep Training and Validation Study With Benchmarking. Gastroenterology.

[31] de Groof A.J., Struyvenberg M.R., Fockens K.N., van der Putten J., van der Sommen F., Boers T.G., Zinger S., Bisschops R., de With P.H., Pouw R.E. (2020). Deep learning algorithm detection of Barrett’s neoplasia with high accuracy during live endoscopic procedures: A pilot study (with video). Gastrointest. Endos.c.

[32] Shiroma S., Yoshio T., Kato Y., Horie Y., Namikawa K., Tokai Y., Yoshimizu S., Yoshizawa N., Horiuchi Y., Ishiyama A. (2021). Ability of artificial intelligence to detect T1 esophageal squamous cell carcinoma from endoscopic videos and the effects of real-time assistance. Sci. Rep..

[33] Yang X.X., Li Z., Shao X.J., Ji R., Qu J.Y., Zheng M.Q., Sun Y.N., Zhou R.C., You H., Li L.X. (2020). Real-time artificial intelligence for endoscopic diagnosis of early esophageal squamous cell cancer (with video). Dig. Endosc..

[34] Bang C.S., Lee J.J., Baik G.H. (2021). Computer-aided diagnosis of esophageal cancer and neoplasms in endoscopic images: A systematic review and meta-analysis of diagnostic test accuracy. Gastrointest. Endosc..

[35] Shin D., Protano M.A., Polydorides A.D., Dawsey S.M., Pierce M.C., Kim M.K., Schwarz R.A., Quang T., Parikh N., Bhutani M.S. (2015). Quantitative analysis of high-resolution microendoscopic images for diagnosis of esophageal squamous cell carcinoma. Clin. Gastroenterol. Hepatol..

[36] Quang T., Schwarz R.A., Dawsey S.M., Tan M.C., Patel K., Yu X., Wang G., Zhang F., Xu H., Anandasabapathy S. (2016). A tablet-interfaced high-resolution microendoscope with automated image interpretation for real-time evaluation of esophageal squamous cell neoplasia. Gastrointest. Endosc..

[37] van der Sommen F., Zinger S., Curvers W.L., Bisschops R., Pech O., Weusten B.L., Bergman J.J.G.H.M., de With P.H.N., Schoon E.J. (2016). Computer-aided detection of early neoplastic lesions in Barrett’s esophagus. Endoscopy.

[38] Mendel R., Ebigbo A., Probst A., Messmann H., Palm C. (2017). Barrett’s Esophagus Analysis Using Convolutional Neural Networks. Bildverarbeitung für die Medizin.

[39] Ebigbo A., Mendel R., Probst A., Manzeneder J., Souza L.A., Papa J.P., Palm C., Messmann H. (2019). Computer-aided diagnosis using deep learning in the evaluation of early oesophageal adenocarcinoma. Gut.

[40] Zhao Y.Y., Xue D.X., Wang Y.L., Zhang R., Sun B., Cai Y.P., Feng H., Cai Y., Xu J.-M. (2019). Computer-assisted diagnosis of early esophageal squamous cell carcinoma using narrow-band imaging magnifying endoscopy. Endoscopy.

[41] Nakagawa K., Ishihara R., Aoyama K., Ohmori M., Nakahira H., Matsuura N., Shichijo S., Nishida T., Yamada T., Yamaguchi S. (2019). Classification for invasion depth of esophageal squamous cell carcinoma using a deep neural network compared with experienced endoscopists. Gastrointest. Endosc..

[42] Guo L., Xiao X., Wu C., Zeng X., Zhang Y., Du J., Bai S., Xie J., Zhang Z., Li Y. (2020). Real-time automated diagnosis of precancerous lesions and early esophageal squamous cell carcinoma using a deep learning model (with videos). Gastrointest. Endosc..

[43] Hashimoto R., Requa J., Dao T., Ninh A., Tran E., Mai D., Lugo M., El-Hage Chehade N., Chang K.J., Karnes W.E. (2020). Artificial intelligence using convolutional neural networks for real-time detection of early esophageal neoplasia in Barrett’s esophagus (with video). Gastrointest. Endosc..

[44] Ohmori M., Ishihara R., Aoyama K., Nakagawa K., Iwagami H., Matsuura N., Shichiji S., Yamamoto K., Nagaike K., Nakahara M. (2020). Endoscopic detection and differentiation of esophageal lesions using a deep neural network. Gastrointest. Endosc..

[45] Li B., Cai S.L., Tan W.M., Li J.C., Yalikong A., Feng X.S., Yu H.H., Lu P.X., Feng Z., Yao L.Q. (2021). Comparative study on artificial intelligence systems for detecting early esophageal squamous cell carcinoma between narrow-band and white-light imaging. World J. Gastroenterol..

[46] Ebigbo A., Mendel R., Rückert T., Schuster L., Probst A., Manzeneder J., Prinz F., Mende M., Steinbrück I., Faiss S. (2021). Endoscopic prediction of submucosal invasion in Barrett’s cancer with the use of artificial intelligence: A pilot study. Endoscopy.

[47] Colom R., Karama S., Jung R.E., Haier R.J. (2010). Human intelligence and brain networks. Dialogues Clin. Neurosci..

[48] Li L., Chen Y., Shen Z., Zhang X., Sang J., Ding Y., Yang X., Li J., Chen M., Jin C. (2020). Convolutional neural network for the diagnosis of early gastric cancer based on magnifying narrow band imaging. Gastric Cancer.

[49] Goodwin C.S. (1997). Helicobacter pylori gastritis, peptic ulcer, and gastric cancer: Clinical and molecular aspects. Clin. Infect. Dis..

[50] Huang C.R., Sheu B.S., Chung P.C., Yang H.B. (2004). Computerized diagnosis of Helicobacter pylori infection and associated gastric inflammation from endoscopic images by refined feature selection using a neural network. Endoscopy.

[51] Shichijo S., Nomura S., Aoyama K., Nishikawa Y., Miura M., Shinagawa T., Takiyama H., Tanimoto T., Ishihara S., Matsuo K. (2017). Application of Convolutional Neural Networks in the Diagnosis of Helicobacter pylori Infection Based on Endoscopic Images. EBioMedicine.

[52] Zheng W., Zhang X., Kim J.J., Zhu X., Ye G., Ye B., Wang J., Luo S., Li J., Yu T. (2019). High Accuracy of Convolutional Neural Network for Evaluation of Helicobacter pylori Infection Based on Endoscopic Images: Preliminary Experience. Clin. Transl. Gastroenterol..

[53] Nakashima H., Kawahira H., Kawachi H., Sakaki N. (2018). Artificial intelligence diagnosis of Helicobacter pylori infection using blue laser imaging-bright and linked color imaging: A single-center prospective study. Ann. Gastroenterol..

[54] Bang C.S., Lee J.J., Baik G.H. (2020). Artificial Intelligence for the Prediction of Helicobacter Pylori Infection in Endoscopic Images: Systematic Review and Meta-Analysis Of Diagnostic Test Accuracy. J. Med. Internet Res..

[55] Japanese Gastric Cancer Association (2021). Japanese gastric cancer treatment guidelines 2018 (5th edition). Gastric Cancer.

[56] Horiuchi Y., Hirasawa T., Ishizuka N., Tokai Y., Namikawa K., Yoshimizu S., Ishiyama A., Yoshio T., Tsuchida T., Fujisaki J. (2020). Performance of a computer-aided diagnosis system in diagnosing early gastric cancer using magnifying endoscopy videos with narrow-band imaging (with videos). Gastrointest. Endosc..

[57] Zhu Y., Wang Q.C., Xu M.D., Zhang Z., Cheng J., Zhong Y.S., Zhang Y.Q., Chen W.F., Yao L.Q., Zhou P.H. (2019). Application of convolutional neural network in the diagnosis of the invasion depth of gastric cancer based on conventional endoscopy. Gastrointest. Endosc..

[58] Cho B.J., Bang C.S., Lee J.J., Seo C.W., Kim J.H. (2020). Prediction of Submucosal Invasion for Gastric Neoplasms in Endoscopic Images Using Deep-Learning. J. Clin. Med..

[59] Ali H., Yasmin M., Sharif M., Rehmani M.H. (2018). Computer assisted gastric abnormalities detection using hybrid texture descriptors for chromoendoscopy images. Comput. Methods Programs Biomed..

[60] Kanesaka T., Lee T.C., Uedo N., Lin K.P., Chen H.Z., Lee J.Y., Wang H.P., Chang H.T. (2018). Computer-aided diagnosis for identifying and delineating early gastric cancers in magnifying narrow-band imaging. Gastrointest. Endosc..

[61] Kailin J., Xiaotao J., Jinglin P., Yi W., Yuanchen H., Senhui W., Shaoyang L., Kechao N., Zhihua Z., Shuling J. (2021). Current Evidence and Future Perspective of Accuracy of Artificial Intelligence Application for Early Gastric Cancer Diagnosis with Endoscopy: A Systematic and Meta-Analysis. Front. Med..

[62] Joo M., Park A., Kim K., Son W.J., Lee H.S., Lim G., Lee J., Lee D.H., An J., Kim J.H. (2019). A Deep Learning Model for Cell Growth Inhibition IC50 Prediction and Its Application for Gastric Cancer Patients. Int. J. Mol. Sci..

[63] Miyaki R., Yoshida S., Tanaka S., Kominami Y., Sanomura Y., Matsuo T., Oka S., Ryetchev B., Tamaki T., Koide T. (2015). A computer system to be used with laser-based endoscopy for quantitative diagnosis of early gastric cancer. J. Clin. Gastroenterol..

[64] Hirasawa T., Aoyama K., Tanimoto T., Ishihara S., Shichijo S., Ozawa T., Ohnishi T., Fujishiro M., Matsuo K., Fujisaki J. (2018). Application of artificial intelligence using a convolutional neural network for detecting gastric cancer in endoscopic images. Gastric. Cancer.

[65] Liu X., Wang C., Hu Y., Zeng Z., Bai J., Liao G. Transfer Learning with Convolutional Neural Network for Early Gastric Cancer Classification on Magnifiying Narrow-Band Imaging Images. Proceedings of the 2018 25th IEEE International Conference on Image Processing (ICIP).

[66] Horiuchi Y., Aoyama K., Tokai Y., Hirasawa T., Yoshimizu S., Ishiyama A., Yoshio T., Tsuchida T., Fujisaki J., Tada T. (2020). Convolutional Neural Network for Differentiating Gastric Cancer from Gastritis Using Magnified Endoscopy with Narrow Band Imaging. Dig. Dis. Sci..

[67] Guimaraes P., Keller A., Fehlmann T., Lammert F., Casper M. (2020). Deep-learning based detection of gastric precancerous conditions. Gut.

[68] Yasuda T., Hiroyasu T., Hiwa S., Okada Y., Hayashi S., Nakahata Y., Yasuda Y., Omatsu T., Obora A., Kojima T. (2020). Potential of automatic diagnosis system with linked color imaging for diagnosis of Helicobacter pylori infection. Dig. Endosc..

[69] Wu L., He X., Liu M., Xie H., An P., Zhang J., Zhang H., Ai Y., Tong Q., Guo M. (2021). Evaluation of the effects of an artificial intelligence system on endoscopy quality and preliminary testing of its performance in detecting early gastric cancer: A randomized controlled trial. Endoscopy.

[70] Xia J., Xia T., Pan J., Gao F., Wang S., Qian Y.Y., Wang H., Zhao J., Jiang X., Zou W.-B. (2021). Use of artificial intelligence for detection of gastric lesions by magnetically controlled capsule endoscopy. Gastrointest. Endosc..

[71] Institute NC. Surveillance Epidemiology and End Results (SEER) database National Cancer Institute (NCI). https://seer.cancer.gov/statfacts/html/colorect.html..

[72] Zauber A.G., Winawer S.J., O’Brien M.J., Lansdorp-Vogelaar I., van Ballegooijen M., Hankey B.F., Shi W., Bond J.H., Schapiro M., Panish J.F. (2012). Colonoscopic Polypectomy and Long-Term Prevention of Colorectal-Cancer Deaths. N. Engl. J. Med..

[73] Pannala R., Krishnan K., Melson J., Parsi M.A., Schulman A.R., Sullivan S., Trikudanathan G., Trindade A.J., Watson R.R., Maple J.T. (2020). Artificial intelligence in gastrointestinal endoscopy. VideoGIE.

[74] Nazarian S., Glover B., Ashrafian H., Darzi A., Teare J. (2021). Diagnostic Accuracy of Artificial Intelligence and Computer-Aided Diagnosis for the Detection and Characterization of Colorectal Polyps: Systematic Review and Meta-analysis. J. Med. Internet Res..

[75] Robertson A.R., Segui S., Wenzek H., Koulaouzidis A. (2021). Artificial intelligence for the detection of polyps or cancer with colon capsule endoscopy. Ther Adv. Gastrointest. Endosc..

[76] Laiz P., Vitrià J., Wenzek H., Malagelada C., Azpiroz F., Seguí S. (2020). WCE polyp detection with triplet based embeddings. Comput. Med. Imaging Graph..

[77] Tischendorf J.J., Gross S., Winograd R., Hecker H., Auer R., Behrens A., Trautwein C., Aach T., Stehle T. (2010). Computer-aided classification of colorectal polyps based on vascular patterns: A pilot study. Endoscopy.

[78] Takemura Y., Yoshida S., Tanaka S., Kawase R., Onji K., Oka S., Tamaki T., Tyetchev B., Kaneda K., Yoshihara M. (2012). Computer-aided system for predicting the histology of colorectal tumors by using narrow-band imaging magnifying colonoscopy (with video). Gastrointest. Endosc..

[79] Mori Y., Kudo S.E., Wakamura K., Misawa M., Ogawa Y., Kutsukawa M., Kudo T., Hayashi T., Miyachi H., Ishida F. (2015). Novel computer-aided diagnostic system for colorectal lesions by using endocytoscopy (with videos). Gastrointest. Endosc..

[80] Fernández-Esparrach G., Bernal J., López-Cerón M., Córdova H., Sánchez-Montes C., Rodríguez de Miguel C., Javier Sanchez F. (2016). Exploring the clinical potential of an automatic colonic polyp detection method based on the creation of energy maps. Endoscopy.

[81] Kominami Y., Yoshida S., Tanaka S., Sanomura Y., Hirakawa T., Raytchev B., Tamaki T., Koide T., Kaneda K., Chayama K. (2016). Computer-aided diagnosis of colorectal polyp histology by using a real-time image recognition system and narrow-band imaging magnifying colonoscopy. Gastrointest. Endosc..

[82] Sun Young P., Dusty S. (2016). Colonoscopic polyp detection using convolutional neural networks. ProcSPIE.

[83] Tamai N., Saito Y., Sakamoto T., Nakajima T., Matsuda T., Sumiyama K., Tajire H., Koyama R., Kido S. (2017). Effectiveness of computer-aided diagnosis of colorectal lesions using novel software for magnifying narrow-band imaging: A pilot study. Endosc. Int. Open..

[84] Zhang R., Zheng Y., Mak T.W., Yu R., Wong S.H., Lau J.Y., Poon C.C.Y. (2017). Automatic Detection and Classification of Colorectal Polyps by Transferring Low-Level CNN Features From Nonmedical Domain. IEEE J. Biomed. Health Inform..

[85] Misawa M., Kudo S.E., Mori Y., Cho T., Kataoka S., Yamauchi A., Ogawa Y., Maeda Y., Takeda K., Ichimasa K. (2018). Artificial Intelligence-Assisted Polyp Detection for Colonoscopy: Initial Experience. Gastroenterology.

[86] Mori Y., Kudo S.E., Misawa M., Saito Y., Ikematsu H., Hotta K., Ohtsuka K., Urushibara F., Kataoka S., Ogawa Y. (2018). Real-Time Use of Artificial Intelligence in Identification of Diminutive Polyps During Colonoscopy: A Prospective Study. Ann. Intern. Med..

[87] Urban G., Tripathi P., Alkayali T., Mittal M., Jalali F., Karnes W., Baldi P. (2018). Deep Learning Localizes and Identifies Polyps in Real Time With 96% Accuracy in Screening Colonoscopy. Gastroenterology.

[88] Byrne M.F., Chapados N., Soudan F., Oertel C., Linares Pérez M., Kelly R., Iqbal N., Chandelier F., Rex D.K. (2019). Real-time differentiation of adenomatous and hyperplastic diminutive colorectal polyps during analysis of unaltered videos of standard colonoscopy using a deep learning model. Gut.

[89] Figueiredo P.N., Figueiredo I.N., Pinto L., Kumar S., Tsai Y.-H.R., Mamonov A.V. (2019). Polyp detection with computer-aided diagnosis in white light colonoscopy: Comparison of three different methods. Endosc. Int. Open..

[90] Horiuchi H., Tamai N., Kamba S., Inomata H., Ohya T.R., Sumiyama K. (2019). Real-time computer-aided diagnosis of diminutive rectosigmoid polyps using an auto-fluorescence imaging system and novel color intensity analysis software. Scand. J. Gastroenterol..

[91] Ito N., Kawahira H., Nakashima H., Uesato M., Miyauchi H., Matsubara H. (2019). Endoscopic Diagnostic Support System for cT1b Colorectal Cancer Using Deep Learning. Oncology.

[92] Wang P., Berzin T.M., Glissen Brown J.R., Bharadwaj S., Becq A., Xiao X., Liu P., Li L., Song Y., Zhang D. (2019). Real-time automatic detection system increases colonoscopic polyp and adenoma detection rates: A prospective randomised controlled study. Gut.

[93] Jin E.H., Lee D., Bae J.H., Kang H.Y., Kwak M.S., Seo J.Y., Yang J.I., Yang S.Y., Lim S.H., Yim J.Y. (2020). Improved Accuracy in Optical Diagnosis of Colorectal Polyps Using Convolutional Neural Networks with Visual Explanations. Gastroenterology.

[94] Kudo S.E., Misawa M., Mori Y., Hotta K., Ohtsuka K., Ikematsu H., Saito Y., Takeda K., Nakmura H., Ichimasa K. (2020). Artificial Intelligence-assisted System Improves Endoscopic Identification of Colorectal Neoplasms. Clin. Gastroenterol. Hepatol..

[95] Nakajima Y., Zhu X., Nemoto D., Li Q., Guo Z., Katsuki S., Hayashi Y., Utano K., Aizawa M., Takezawa T. (2020). Diagnostic performance of artificial intelligence to identify deeply invasive colorectal cancer on non-magnified plain endoscopic images. Endosc. Int. Open..

[96] Ozawa T., Ishihara S., Fujishiro M., Kumagai Y., Shichijo S., Tada T. (2020). Automated endoscopic detection and classification of colorectal polyps using convolutional neural networks. Ther. Adv. Gastroenterol..

[97] Repici A., Badalamenti M., Maselli R., Correale L., Radaelli F., Rondonotti E., Ferrara E., Spadaccini M., Alkandari A., Fugazza A. (2020). Efficacy of Real-Time Computer-Aided Detection of Colorectal Neoplasia in a Randomized Trial. Gastroenterology.

[98] Lai L.L., Blakely A., Invernizzi M., Lin J., Kidambi T., Melstrom K.A., Yu K., Lu T. (2021). Separation of color channels from conventional colonoscopy images improves deep neural network detection of polyps. J. Biomed. Opt..

[99] Luo Y., Zhang Y., Liu M., Lai Y., Liu P., Wang Z., Xing T., Huang Y., Li Y., Li A. (2021). Artificial Intelligence-Assisted Colonoscopy for Detection of Colon Polyps: A Prospective, Randomized Cohort Study. J. Gastrointest. Surg..

[100] Kudo S.E., Ichimasa K., Villard B., Mori Y., Misawa M., Saito S., Hotta K., Saito Y., Matsuda T., Yamada K. (2021). Artificial Intelligence System to Determine Risk of T1 Colorectal Cancer Metastasis to Lymph Node. Gastroenterology.

[101] Ichimasa K., Kudo S.E., Mori Y., Misawa M., Matsudaira S., Kouyama Y., Baba T., Hidaka E., Wakamura K., Hayashi T. (2018). Correction: Artificial intelligence may help in predicting the need for additional surgery after endoscopic resection of T1 colorectal cancer. Endoscopy.

[102] Lippi G., Mattiuzzi C. (2020). The global burden of pancreatic cancer. Arch. Med. Sci..

[103] Laoveeravat P.A.P., Brenner A.R., Gabr M.M., Habr F.G., Atsawarungruangkit A. (2021). Artificial intelligence for pancreatic cancer detection: Recent development and future direction. Artif. Intell. Gastroenterol..

[104] Liu S.L., Li S., Guo Y.T., Zhou Y.P., Zhang Z.D., Li S., Lu Y. (2019). Establishment and application of an artificial intelligence diagnosis system for pancreatic cancer with a faster region-based convolutional neural network. Chin. Med. J..

[105] Li H., Shi K., Reichert M., Lin K., Tselousov N., Braren R., Fu D., Schmid R., Li J., Menze B. (2019). Differential Diagnosis for Pancreatic Cysts in CT Scans Using Densely-Connected Convolutional Networks. Annu. Int. Conf. IEEE Eng Med. Biol. Soc..

[106] Chu L.C., Park S., Kawamoto S., Fouladi D.F., Shayesteh S., Zinreich E.S., Graves J.S., Horton K.M., Hruban R.H., Yuille A.L. (2019). Utility of CT Radiomics Features in Differentiation of Pancreatic Ductal Adenocarcinoma from Normal Pancreatic Tissue. AJR Am. J. Roentgenol..

[107] Wei R., Lin K., Yan W., Guo Y., Wang Y., Li J., Zhu J. (2019). Computer-Aided Diagnosis of Pancreas Serous Cystic Neoplasms: A Radiomics Method on Preoperative MDCT Images. Technol. Cancer Res. Treat..

[108] Li S., Jiang H., Wang Z., Zhang G., Yao Y.D. (2018). An effective computer aided diagnosis model for pancreas cancer on PET/CT images. Comput. Methods Programs Biomed..

[109] Corral J.E., Hussein S., Kandel P., Bolan C.W., Bagci U., Wallace M.B. (2019). Deep Learning to Classify Intraductal Papillary Mucinous Neoplasms Using Magnetic Resonance Imaging. Pancreas.

[110] Kaissis G.A., Ziegelmayer S., Lohöfer F.K., Harder F.N., Jungmann F., Sasse D., Muckenhuber A., Yen H.-Y., Steiger K., Siveke J. (2020). Image-Based Molecular Phenotyping of Pancreatic Ductal Adenocarcinoma. J. Clin. Med..

[111] Ozkan M., Cakiroglu M., Kocaman O., Kurt M., Yilmaz B., Can G., Korkmaz U., Dandil E., Eksi Z. (2016). Age-based computer-aided diagnosis approach for pancreatic cancer on endoscopic ultrasound images. Endosc. Ultrasound.

[112] Marya N.B., Powers P.D., Chari S.T., Gleeson F.C., Leggett C.L., Abu Dayyeh B.K., Chandrasekhara V., Iyer P.G., Majumder S., Pearson R.K. (2021). Utilisation of artificial intelligence for the development of an EUS-convolutional neural network model trained to enhance the diagnosis of autoimmune pancreatitis. Gut.

[113] Tonozuka R., Itoi T., Nagata N., Kojima H., Sofuni A., Tsuchiya T., Ishii K., Tanaka R., Nagakawa Y., Mukai S. (2021). Deep learning analysis for the detection of pancreatic cancer on endosonographic images: A pilot study. J. Hepatobiliary Pancreat Sci..

[114] Săftoiu A., Vilmann P., Gorunescu F., Gheonea D.I., Gorunescu M., Ciurea T., Popescu G.L., Iordache A., Hassan H., Iordache S. (2008). Neural network analysis of dynamic sequences of EUS elastography used for the differential diagnosis of chronic pancreatitis and pancreatic cancer. Gastrointest. Endosc..

[115] Zhang M.M., Yang H., Jin Z.D., Yu J.G., Cai Z.Y., Li Z.S. (2010). Differential diagnosis of pancreatic cancer from normal tissue with digital imaging processing and pattern recognition based on a support vector machine of EUS images. Gastrointest. Endosc..

[116] Săftoiu A., Vilmann P., Gorunescu F., Janssen J., Hocke M., Larsen M., Iglesias-Garcia J., Arcidiacono P., Will U., Giovannini M. (2012). Efficacy of an artificial neural network-based approach to endoscopic ultrasound elastography in diagnosis of focal pancreatic masses. Clin. Gastroenterol. Hepatol..

[117] Săftoiu A., Vilmann P., Dietrich C.F., Iglesias-Garcia J., Hocke M., Seicean A., Ignee A., Hassan H., Streba C.T., Ioncică A.M. (2015). Quantitative contrast-enhanced harmonic EUS in differential diagnosis of focal pancreatic masses (with videos). Gastrointest. Endosc..

[118] Kuwahara T., Hara K., Mizuno N., Okuno N., Matsumoto S., Obata M., Kurita Y., Koda H., Toriyama K., Onishi S. (2019). Usefulness of Deep Learning Analysis for the Diagnosis of Malignancy in Intraductal Papillary Mucinous Neoplasms of the Pancreas. Clin. Transl. Gastroenterol..

[119] Goggins M. (2005). Molecular markers of early pancreatic cancer. J. Clin. Oncol..

[120] Macha M.A., Seshacharyulu P., Krishn S.R., Pai P., Rachagani S., Jain M., Batra S.K. (2014). MicroRNAs (miRNAs) as biomarker(s) for prognosis and diagnosis of gastrointestinal (GI) cancers. Curr. Pharm. Des..

[121] Yan Q., Hu D., Li M., Chen Y., Wu X., Ye Q., Wang Z., He L., Zhu J. (2020). The Serum MicroRNA Signatures for Pancreatic Cancer Detection and Operability Evaluation. Front. Bioeng. Biotechnol..

[122] Alizadeh Savareh B., Asadzadeh Aghdaie H., Behmanesh A., Bashiri A., Sadeghi A., Zali M., Shams R. (2020). A machine learning approach identified a diagnostic model for pancreatic cancer through using circulating microRNA signatures. Pancreatology.

[123] Sinkala M., Mulder N., Martin D. (2020). Machine Learning and Network Analyses Reveal Disease Subtypes of Pancreatic Cancer and their Molecular Characteristics. Sci. Rep..

[124] (NCI) NCI Surveillance Epidemiology and End Results (SEER) database. https://seer.cancer.gov/statfacts/html/livibd.html..

[125] Virmani J., Kumar V., Kalra N., Khandelwal N. (2013). SVM-based characterization of liver ultrasound images using wavelet packet texture descriptors. J. Digit. Imaging.

[126] Wu C.-C., Lee W.-L., Chen Y.-C., Lai C.-H., Hsieh K.-S. (2012). Ultrasonic liver tissue characterization by feature fusion. Expert Syst. Appl..

[127] Lee W.-L. (2013). An ensemble-based data fusion approach for characterizing ultrasonic liver tissue. Appl. Soft Comput..

[128] Bharti P., Mittal D., Ananthasivan R. (2018). Preliminary Study of Chronic Liver Classification on Ultrasound Images Using an Ensemble Model. Ultrason. Imaging.

[129] Schmauch B., Herent P., Jehanno P., Dehaene O., Saillard C., Aubé C., Luciani A., Lassau N., Jégou S. (2019). Diagnosis of focal liver lesions from ultrasound using deep learning. Diagn. Interv. Imaging.

[130] Cao S.-E., Zhang L.-Q., Kuang S.-C., Shi W.-Q., Hu B., Xie S.-D., Chen Y.-N., Liu H., Chen S.-M., Jiang T. (2020). Multiphase convolutional dense network for the classification of focal liver lesions on dynamic contrast-enhanced computed tomography. World J. Gastroenterol..

[131] Yasaka K., Akai H., Abe O., Kiryu S. (2018). Deep Learning with Convolutional Neural Network for Differentiation of Liver Masses at Dynamic Contrast-enhanced CT: A Preliminary Study. Radiology.

[132] Hamm C.A., Wang C.J., Savic L.J., Ferrante M., Schobert I., Schlachter T., Lin M., Duncan J.S., Weinreb J.C., Chapiro J. (2019). Deep learning for liver tumor diagnosis part I: Development of a convolutional neural network classifier for multi-phasic MRI. Eur. Radiol..

[133] Oestmann P.M., Wang C.J., Savic L.J., Hamm C.A., Stark S., Schobert I., Gebauer B., Schlachter T., Lin M., Weinreb J.C. (2021). Deep learning-assisted differentiation of pathologically proven atypical and typical hepatocellular carcinoma (HCC) versus non-HCC on contrast-enhanced MRI of the liver. Eur. Radiol..

[134] Qu J., Hiruta N., Terai K., Nosato H., Murakawa M., Sakanashi H. (2018). Gastric Pathology Image Classification Using Stepwise Fine-Tuning for Deep Neural Networks. J. Healthc. Eng..

[135] Sharma H., Zerbe N., Klempert I., Hellwich O., Hufnagl P. (2017). Deep convolutional neural networks for automatic classification of gastric carcinoma using whole slide images in digital histopathology. Comput. Med. Imaging Graph..

[136] Iizuka O., Kanavati F., Kato K., Rambeau M., Arihiro K., Tsuneki M. (2020). Deep learning models for histopathological classification of gastric and colonic epithelial tumours. Sci. Rep..

[137] Momeni-Boroujeni A., Yousefi E., Somma J. (2017). Computer-assisted cytologic diagnosis in pancreatic FNA: An application of neural networks to image analysis. Cancer Cytopathol..

[138] Yu C., Helwig E.J. (2021). Artificial intelligence in gastric cancer: A translational narrative review. Ann. Transl. Med..

